# Photosynthetic gas exchange responses of *Swietenia macrophylla* King and *Melia azedarach* L. plantations under drought conditions

**DOI:** 10.1186/s40529-017-0212-8

**Published:** 2017-12-02

**Authors:** Hong-Chyi Jhou, Ya-Nan Wang, Chung-Shien Wu, Jui-Chu Yu, Chung-I Chen

**Affiliations:** 1 The Experimental Forest, National Taiwan University, 55750 Nantou, Taiwan; 2 Department of Forestry and Resource Conservation, National Taiwan University, 10617 Taipei, Taiwan; 3 Biodiversity Research Center, Academia Sinica, 11529 Taipei, Taiwan

**Keywords:** Photosynthesis, Prolonged drought, Soil water content, Stomatal conductance, Transpiration rate, Vapor pressure deficit

## Abstract

**Background:**

The environmental stresses caused by climate change have become more severe in recent decades, affecting tree growth and physiology. Tropical forests have great potential for global carbon sequestration. However, they suffer from heavy rainfall and prolonged dry periods due to climate change. *Swietenia macrophylla* King and *Melia azedarach* L. are economically valuable trees that are widely planted in southern Taiwan. Plantations are exposed to either prolonged dry periods or heavy rainfall within the seasons of tropical monsoon areas. Photo-physiological comparisons may provide information that can improve management of *S. macrophylla* and *M. azedarach* plantations in tropical regions.

**Results:**

Both species exhibited a midday depression in leaf photosynthesis regardless of the season. The net photosynthetic rate (*P*_N_), stomatal conductance (*g*_s_), and transpiration rate (*E*) in the dry season all significantly decreased in both tree species. In addition, *M. azedarach* used water more efficiently than did *S. macrophylla* during the dry season, but *S. macrophylla* had higher *P*_N_ compared with that in *M. azedarach* during the wet season. Temperature and vapor pressure deficit influenced *P*_N_ variation in *S. macrophylla* and *M. azedarach*, respectively. Our data suggested that the *P*_N_ and *g*_s_ of *M. azedarach*, but not of *S. macrophylla*, were linearly correlated during the dry season. The reduction of the leaf area was more sever in *M. azedarach* than in *S. macrophylla*, thus preventing water loss more efficiently.

**Conclusions:**

*M. azedarach* adapted to drought by reducing total leaf area and maintaining higher *P*_N_, *g*_s_, *E*, and WUE compared with those measured in *S. macrophylla* during the dry season. *M. azedarach* is more drought adaptation and more suitable for both humid and semi-humid areas than *S. macrophylla*, whereas the latter should be limited to more humid areas.

## Background

Global climate change has increased the incidence and severity of extreme climate events such as droughts and high temperatures (IPCC 2015), which strongly affect plant physiology and growth. Numerous reports have emphasized that water stress negatively affects gas exchange, which often results in lower leaf photosynthesis, stomata conductance, and transpiration (Ogaya and Peñuelas 2003; Leuzinger et al. 2005; Guerfel et al. 2009; Li et al. 2016). Decreased leaf photosynthesis can be caused by either stomatal or non-stomatal limitations (Taiz and Zeiger 2002). Therefore, the measurement of gas exchange in plants could reflect the physiological response to environmental conditions and the relationship between gas exchange and environmental variables could provide a more detailed understanding of how environmental variables limit photosynthesis, growth performance, and biomass (Ishida et al. 1996).

Tropical forests play an important role in the global carbon cycle because they account for a large part of terrestrial net primary productivity, while covering a lesser part of the global land surface (Slot and Winter 2017). Tropical areas experience wet seasons (in the summer) with heavy rainfall and prolonged dry seasons (in the winter), which can strongly affect plant distribution and growth (Engelbrecht et al. 2006). Taiwan’s subtropical climate is characterized by an average annual rainfall of > 2500 mm, with heterogeneous distribution across the seasons and geographical areas. In particular, southern Taiwan has had increasingly frequent high temperatures and rainstorms followed by extended droughts. These conditions greatly affect tree growth in the area. To better understand how these changing environmental factors affect plant growth in this region, we estimated gas-exchange variables in two Meliaceae tree species that are widely planted in southern Taiwan: *Swietenia macrophylla* King and *Melia azedarach* L. The semi-deciduous *S. macrophylla* originated from Central and South America, and it is a major forestation species of high economic value in the Taiwanese lumber industry (Grogan et al. 2005). Although generally characterized by shade intolerance, *S. macrophylla* seedlings are able to grow in the forest understory (Grogan et al. 2005; Cordeiro et al. 2009). The deciduous *M. azedarach*, a native to Taiwan and South China, is fast-growing and shade-intolerant, with leaves and fruits that contain insecticidal compounds. The species is also harvested for its high-quality wood (Kuo et al. 2004; Liao et al. 2004), making it a popular species to cultivate. Both species have shallow root systems that are characteristic of many tropical trees (Toky and Bisht 1992; Dünisch et al. 2003).

Several researches demonstrated that different species adopt different strategies in response to seasonal environmental conditions (Sun et al. 2011; Koller et al. 2013; Arndt et al. 2015). For example, gas exchange rate, water use efficiency, chlorophyll fluorescence and seasonal defoliation fluctuate among seasons and species. Thus, preliminary observations revealed that both species exhibited stronger growth than other species in the study site in southern Taiwan (Chen et al. 2013, 2016a), but still showed a poor growth performance compared with those cultivated in other regions in Taiwan, Brazil, and China. The aim of the present study was to assess the vulnerability of both commercial species to environmental stresses. We hypothesized that the two species may adopt different strategies to adapt to water stress that affect the growth and physiology of plantations under field conditions. Rather than performing a single, instantaneous measurement, we chose to chart the daily and seasonal variation in gas exchange because time-course data provide more reliable information on photosynthetic productivity (Yin et al. 2006). Our results will contribute information that can improve management of *S. macrophylla* and *M. azedarach* plantations in tropical regions.

## Methods

### Plant material and growing conditions

The experimental plantation used for this study is located on the Wan-Long Farm, which belongs to the Pingtung operation branch, Taiwan Sugar Corporation in Sinpi Township, Pingtung, Taiwan (120°36′30″ E, 22°31′26″ N; 69 m above sea level). The total area of the plantation in the farm is approximately 291 ha, and the soil consists of a sandy loam. The study site experiences a typical tropical monsoon climate, with a high frequency of typhoons and late thundershowers in the summer and little precipitation in other seasons. The mean monthly daytime air temperatures for March, June, September, and December in 2010 were 25.1, 29.1, 28.6, and 23.1 °C, respectively; they were measured at a meteorological station located 50 m west of the experimental site. The annual precipitation from 2005 to 2010 was estimated to be 2300–3800 mm, with the majority of the rainfall concentrated in the months between May and September. For example, 92% of the total annual precipitation in 2010 happened between May and September, and the dry season lasted for over half a year (accumulated rainfall between October 2009 and April 2010 was only ~ 70 mm). Based on the climate diagrams of this site, the wet season was defined to last from May to September and the dry season in the remaining months.

At the experimental plantation, 14 broadleaved tree species were planted between 2002 and 2005, and *S. macrophylla* and *M. azedarach* were planted in 2002. Irrigation and fertilization were applied in the first few years after afforestation. Additionally, pruning and mowing were also applied annually in late summer. Generally, *M. azedarach* defoliates in February and sprouts new leaves and buds in March, whereas *S. macrophylla* defoliates partially in March. The trees were planted in the same plot, which was subdivided by species. For the experiment, we selected trees located at a subplot boundary so that the two species were side-by-side with 2.5 m distance between each other. The average diameter at breast height (DBH) and total height of *S. macrophylla* and *M. azedarach* in the experimental plot are presented in Table 1. In 2009, *S. macrophylla* and *M. azedarach* stand densities were 1111 and 1420/ha, and in 2010, they were 1070 and 1413/ha, respectively. The survival rates of *S. macrophylla* and *M. azedarach* on the plantation were 96.3 and 99.5%, respectively.

**Table 1:** Average values (± standard error) of diameter at breast height (DBH), height (H), and number of samples (N) for *Swietenia macrophylla* and *Melia azedarach* in 2010

	*S. macrophylla*	*M. azedarach*
DBH (cm)	9.90 ± 2.88b	11.86 ± 4.14a
H (m)	9.12 ± 1.96a	9.43 ± 1.90a
N	69	104

Means followed by different lowercase letters differ significantly between seasons at *P* < 0.05

Leaf area index (LAI) is a dimensionless quantity variable of the forest canopy defined as the one-sided leaf surface area per ground surface area. Monthly measurements of the LAI per species were taken simultaneously for the top and under canopy of three sample trees at dusk using a plant canopy analyzer (LAI-2200, LI-COR, Lincoln, NE, USA).

### Measurement of gas-exchange variables

Diurnal gas exchange was measured once a month in 2010. We measured a group of ambient variables [photosynthetic photon flux densities (PPFD), leaf-air vapor pressure deficit (VPD_l_), leaf temperature (*T*_l_), ambient CO_2_ concentration (CO_2_)] that could influence diurnal gas exchange and estimated physiological variables [net photosynthetic rate (*P*_N_), stomatal conductance (*g*_s_), transpiration rate (*E*), intercellular CO_2_ concentration (*C*_i_), water use efficiency (WUE)] on four dates within a typical season during 2010: late dry season (27 March), early wet season (26 June), late wet season (25 September), and early dry season (30 December). Data were recorded in the field using two portable photosynthesis systems (LI-6400-sun-sky, LI-COR, USA). For all measurements, the leaves were placed in in the chamber (LI-6400-08), using a clump-on leaf cuvette; the chamber was covered with transparent film at the top to ensure that ambient light of equal intensity reached the leaves. WUE was estimated as *P*_N_/*E*.

Gas-exchange variables were measured in three fully expanded leaves per sampled tree under direct sunlight each hour from 08:00 to 16:00, on the same day in the three sampled trees per species. Because of the limited time available for each measurement, we sampled three nearby trees per species in the same plot. The ambient air temperature was recorded during each sampling, as was the ambient CO_2_, which was approximately 394, 376, 389, and 395 ppm on March 27, June 26, September 25, and December 30, 2010, respectively. The average daytime PPFD above canopy was 1739 ± 667, 1762 ± 800, 1520 ± 856, and 1413 ± 528 µmol photons/m^2^/s in March, June, September, and December, 2010, respectively. The light saturation points of *S. macrophylla* during winter, spring, summer, and autumn were 500, 750, 1000, and 500 µmol photons/m^2^/s, respectively, and in *M. azedarach*, they were 500, 500, 1200, and 500 µmol photons/m^2^/s during the same respective seasons. These values were estimated by measuring the seasonal light response curve in 2011 (data not shown). Soil water content (SWC) and relative humidity (RH) were continuously recorded at a meteorological station 50 m west of the sampled tree plantation using a time-domain reflectometer (CS616; Campbell Scientific Inc., Logan, UT, USA) and a relative humidity probe (HMP45C; Vaisala, Finland). The soil water content device was placed 20 cm underground and the humidity probe was set 9 m aboveground. According to the previous research conducted in this site, the soil profile was shallow (< 40 cm depth) (Cheng et al. 2016). The optimum temperature of photosynthesis is generally between 25 and 30 °C. Thus, we set 30 °C as the break point.

### Data analysis

Collected data were analyzed in *SAS 9.3* (SAS Institute Inc., Cary, NC, USA). Pearson’s correlation was used to examine the relationship between photosynthetic variables and environmental factors. Duncan’s multiple range tests were run a posteriori to test the difference among the seasons and species. Significance was considered at *P* < 0.05 and 0.01. Regression and curve-fitting analyses were performed using SigmaPlot 12.0 (Systat Software Inc, San Jose, CA, USA). The relation among gas exchange parameters was analyzed using the monthly measurement data. Data are presented as mean ± standard error.

## Results

### Environmental conditions

**Fig. 1:**
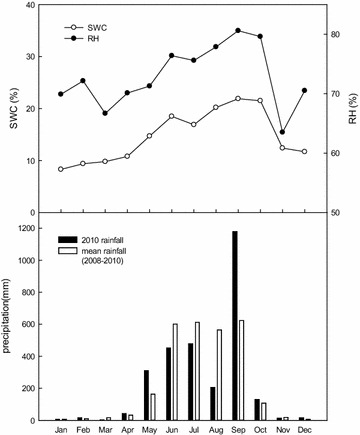
Monthly changes in soil water content (SWC), relative humidity (RH), and rainfall in 2010, and the mean rainfall between 2008 and 2010

The annual rainfall was 2848.5 mm in 2010, and the average annual rainfall was 2764.5 mm during the 2008–2010 period. The recorded rainfall varied considerably in 2010 from a maximum of 1178.5 mm in September to a minimum of 2.5 mm in March. A typhoon was the primary cause of the high rainfall volume in September. The accumulated rainfall from May to September accounted for more than 92% of the annual precipitation, confirming that these months constituted the wet season (Fig. 1). In addition, the fluctuations in SWC and RH also reflected the rainfall volume. Although the precipitation was mainly concentrated between May and September, the SWC and RH were increased from May to October represented the pattern of time lag. For example, both SWC and RH were the highest in September but then decreased drastically in November and December when the precipitation was approximately zero.

**Fig. 2:**
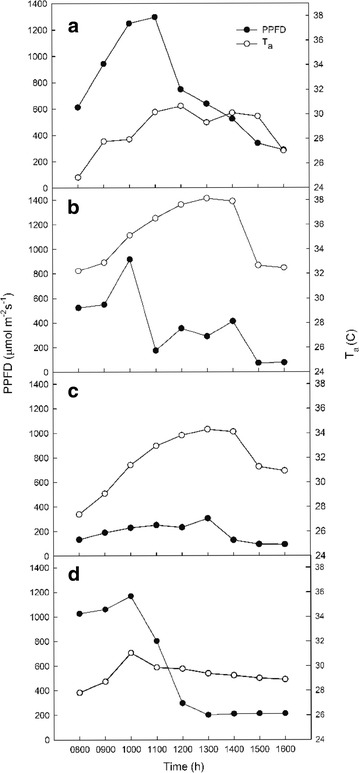
Diurnal variation in photosynthetic photon flux density (PPFD) and air temperature (*T*_a_) in 2010: late dry season (27 March; **a**), early wet season (26 June; **b**), late wet season (25 September; **c**), and early dry season (30 December; **d**)

Across all seasons, PPFD gradually increased throughout the morning and then decreased after noon. March and December air temperatures (*T*_a_) peaked at 30.7 °C (12:00) and 31.1 °C (10:00), respectively. The highest *T*_a_ of the year (38.1 °C) occurred in June at 13:00, but the highest PPFD was measured in September at 13:00, when the maximum *T*_a_ was 34.3 °C (Fig. 2). The highest VPD_a_ for each season was 3.31 kPa at 11:00, 4.22 kPa at 11:00, 3.88 kPa at 13:00, and 3.38 kPa at 11:00 on 27 March, 26 June, 25 September, and 30 December, respectively.

### Gas exchange in leaves

**Fig. 3:**
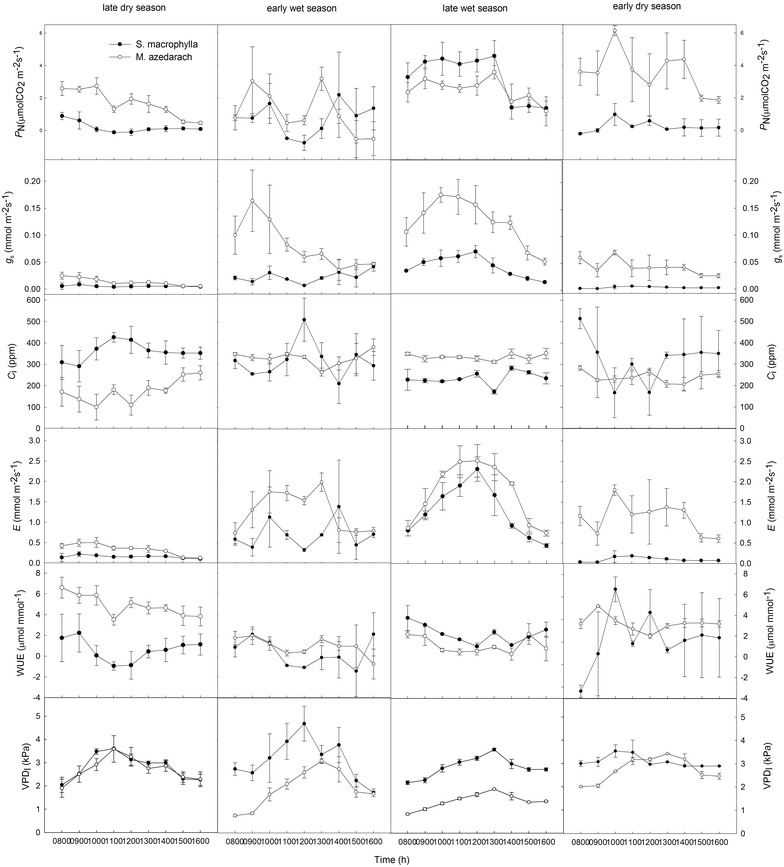
Diurnal variation in gas exchange in *Swietenia macrophylla* and *Melia azedarach* in 2010. Net photosynthetic rate (*P*_N_), stomatal conductance (*g*_s_), intercellular CO_2_ concentration (*C*_i_), water use efficiency (WUE), and leaf-air vapor pressure deficit (VPD_l_), late dry season (27 March), early wet season (26 June), late wet season (25 September), and early dry season (30 December). Data are represented as the mean ± SE (*N* = 3)

Overall, the *g*_s_ and *E* values of both species were higher in the wet season than in the dry season. Interestingly, the two species had distinct *P*_N_ values across all seasons. In the dry season, the *S. macrophylla P*_N_ was low, but in the wet season, it increased by nearly fourfold from 08:00 to 13:00, and then decreased drastically (Fig. 3). In *M. azedarach*, however, the seasonal variation of *P*_N_ was indistinct (Fig. 3). As a result, *M. azedarach* had higher *P*_N_ than *S. macrophylla* in the dry season, but lower *P*_N_ in the wet season.

The two species had similar *g*_s_ values in the dry season, but in the wet season, *M. azedarach* had higher *g*_s_ than *S. macrophylla* throughout the day (Fig. 3). The lowest *g*_s_ values were 0.0041 and 0.0024 mmol H_2_O/m^2^/s in the late dry season and early dry season, respectively. Variation in *C*_i_ was the opposite of *P*_N_, with *S. macrophylla* exhibiting higher *C*_i_ values in the dry season than in the wet season (Fig. 3). A comparison of diurnal WUE revealed that *M. azedarach* had significantly higher values than *S. macrophylla* in the dry season, but the differences between the species were less in the wet season (Fig. 3). The VPD_l_ increased from 8:00 to 11:00 in the dry season, whereas the increase continued until 13:00 in the wet season (Fig. 3). These results show that the gas exchange of both species was representative of different patterns in response to drought conditions.

During the dry season, *P*_N_ had a significantly negative correlation (*P* < 0.01) with C_i_ in both species. Furthermore, in S. *macrophylla*, the *P*_N_ was negatively correlated (*P* < 0.05) with *T*_l_, while in *M. azedarach*, the *P*_N_ value was positively correlated with VPD_l_ and PPFD (*P* < 0.01). During both seasons, the correlation between *g*_s_ and *C*_i_ was significantly positive in *M. azedarach* (*P* < 0.01) but not in *S*. *macrophylla*. During the wet season, *S*. *macrophylla P*_N_ exhibited a significant positive correlation (*P* < 0.01) with *g*_s_ and *E*, but a negative correlation (*P* < 0.01) with *T*_a_ and *T*_l_ (Table 2). There was no significant correlation between the gas exchange variables and CO_2_ in *S*. *macrophylla* regardless of the season. However, in *M. azedarach* the CO_2_ content significantly and positively correlated with g_s_ (*P* < 0.05) and C_i_ (*P* < 0.01) during the dry season, but exhibited a negative correlation with *E* in the wet season (*P* < 0.01).

**Table 2:** Correlation between photosynthetic variables and environmental factors in *Swietenia macrophylla* and *Melia azedarach* during the dry and wet seasons

	Environmental factors^a^								CO_2_
	*g*_s_	*C*_i_	*E*	VPD_a_	VPD_l_	*T*_a_	*T*_1_	PPFD	
Dry season									
*P*_N_	ns/ns	−**/−**	ns/ns	ns/ns	ns/+*	ns/ns	−*/ns	ns/+**	ns/ns
*g*_s_		ns/+**	+**/+**	ns/ns	ns/−**	ns/ns	ns/ns	ns/ns	ns/+*
*C*_i_			ns/ns	ns/−*	ns/−**	ns/ns	ns/ns	ns/−**	ns/+**
*E*				ns/ns	ns/ns	ns/ns	ns/+*	ns/ns	ns/ns
Wet season									
*P*_N_	+**/ns	ns/−*	+**/ns	ns/ns	ns/ns	−**/ns	−**/ns	ns/ns	ns/ns
*g*_s_		ns/+**	+**/+**	ns/−**	ns/−**	ns/ns	−*/+*	ns/ns	ns/ns
*C*_i_			ns/+*	ns/−**	ns/−**	ns/ns	ns/+*	ns/−**	ns/ns
*E*				ns/ns	ns/ns	ns/+**	ns/+**	ns/ns	ns/−**

+ and − symbols represent positive and negative correlation, respectively

ns indicates no significant correlation

*^,^** Indicate significance at *P* < 0.05 and *P* < 0.01, respectively. N = 18

^a^ *g*_s_ (stomatal conductance), *C*_i_ (intercellular CO_2_ concentration), *E* (transpiration rate), VPD_a_ (air vapor pressure deficit), VPD_l_ (leaf-air vapor pressure deficit), *T*_a_ (air temperature), *T*_l_ (leaf temperature), PPFD (photosynthetic photon flux density), CO_2_ (ambient CO_2_ concentration), *S. macrophylla*/*M. azedarach*

Seasonal LAI variation was different between the species (Table 3). LAI was higher in *S. macrophylla* than in *M. azedarach* throughout all seasons. *S. macrophylla* exhibited no significant difference between seasons, although this difference was slightly higher during the wet season. In contrast, *M. azedarach* exhibited a significantly higher LAI in the early wet season than in the late dry season.

**Table 3:** Mean values (± standard error) of leaf area index (LAI) of *Swietenia macrophylla* and *Melia azedarach* in different seasons

Seasons	*S. macrophylla*	*M. azedarach*
Late dry	2.53 ± 0.18a	1.30 ± 0.55b
Early wet	3.21 ± 0.42a	3.04 ± 0.24a
Late wet	3.26 ± 0.96a	2.77 ± 0.77ab
Early dry	2.75 ± 0.19a	1.79 ± 0.18ab

Means followed by different lowercase letters differ significantly between seasons at *P* < 0.05

**Fig. 4:**
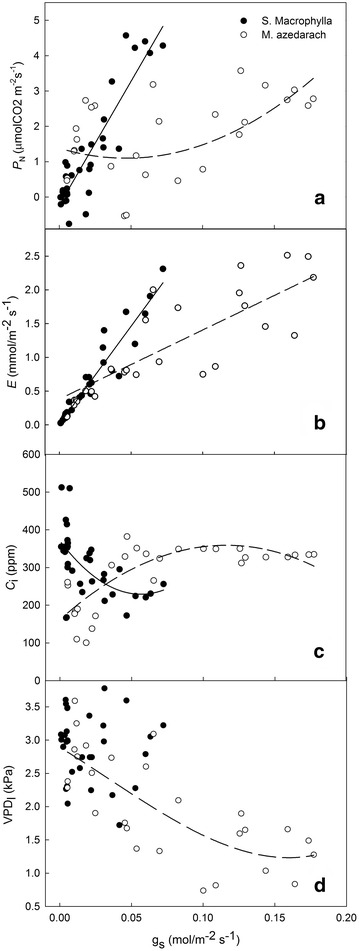
Relationship between stomatal conductance (*g*_s_) and other gas exchange variables in *Swietenia macrophylla* and *Melia azedarach* (*N* = 36). Net photosynthetic rate (*P*_N_; **a**), transpiration (*E*; **b**), intercellular CO_2_ concentration (*C*_i_; **c**), and vapor pressure deficit (VPD_l_; **d**)

Figure 4 depicts the relationship between *g*_s_ and the gas-exchange variables. We observed a linear relationship between *P*_N_ and *g*_s_ in *S. macrophylla*, but a parabolic relationship between these factors in *M. azedarach* (Fig. 4a). The *g*_s_ and *E* values in both species exhibited a significant linear correlation (*P* < 0.01), with the slope for *S. macrophylla* being higher than that for *M. azedarach* (Fig. 4b). The relationship between *g*_s_ and *C*_i_ was distinctly parabolic in both species; in *S. macrophylla* for example, the relationship was a quadratic polynomial, with a decrease in *C*_i_ when *g*_s_ was less than 0.05 mol/m^2^/s^1^ and an increase in *C*_i_ when *g*_s_ was greater than 0.05 mol/m^2^/s. In contrast, *g*_s_ and *C*_i_ in *M. azedarach* were positively correlated under drought conditions, whereas, in the wet season, *C*_i_ remained constant as *g*_s_ increased (Fig. 4c). We observed a curvilinear relationship between *g*_s_ and VPD_l_ in *M. azedarach* (*P* < 0.01), while *g*_s_ decreased sharply when VPD_l_ was over 1.5 kPa. This relationship was not significant in *S. macrophylla* (Fig. 4d).

**Fig. 5:**
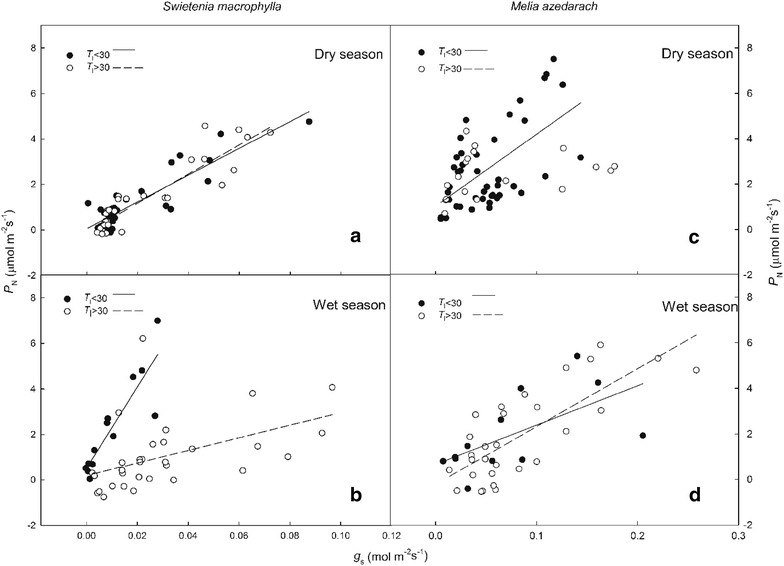
Relationship between *Swietenia macrophylla* and *Melia azedarach* stomatal conductance (*g*_s_) and net photosynthetic rate (*P*_N_) in dry (**a**, **c**) and wet (**b**, **d**) seasons at different leaf temperatures (*T*_l_)

The linear relationship between *P*_N_ and *g*_s_ in both species was divided into two groups by leaf temperatures above and below 30 °C in the dry and wet seasons (Fig. 5). During the dry season, in *S. macrophylla*, a significantly positive linear correlation and similar slope was observed between *P*_N_ and *g*_s_ regardless of the *T*_l_. In the wet season, however, the linear slope at low *T*_l_ was higher than that at high *T*_l_. In contrast, *P*_N_ and *g*_s_ in *M. azedarach* had a similar slope regardless of the *T*_l_ in the wet season.

## Discussion

The influence of many environmental factors on photosynthesis and plant growth have been well researched in recent decades (Colom and Vazzana 2003; Ogaya and Peñuelas 2003; Li and Chen 2009). Zhang et al. (2009) suggested that a cool dry season of 4 months may have long-lasting negative effects on the physiology of Dipterocarpaceae trees in southern China. Similarly, the prolonged drought in 2010 may have influenced the *P*_N_ and *g*_s_ of the trees in our experimental plantation. Gas-exchange dynamics may reflect the ability of a plant to maintain its photosynthetic apparatus under various environmental conditions (Ngugi et al. 2004; Ding et al. 2006). Therefore, we compared diurnal gas-exchange variables across dry and wet seasons in the two tree species.

Midday depression in leaf photosynthesis is common in the canopy leaves of tropical forests which is caused by high temperature, irradiance, or VPD (Ding et al. 2006; Li and Chen 2009; Zhang et al. 2009; Gao et al. 2015). Both species examined in this study exhibited a midday depression in leaf photosynthesis and recovered after 14:00, regardless of the season. These patterns indicate that environmental factors may limit photosynthesis in tropical forests during the midday period, which is consistent with the observations in other evergreen communities during seasonal drought (Koch et al. 1994). Under typical conditions, photosynthetic rates usually increase with PPFD, but in both seasons in our study, the *P*_N_ in *S. macrophylla* was negatively correlated (*P* < 0.01) with *T*_l_ and not correlated with PPFD. However, temperature directly influenced VPD which is an important environment factor affected the leaf gas exchange. Previous estimations based on gas-exchange in peach-palm trees under subtropical conditions indicated that temperature and VPD have stronger effects on *P*_N_ compared with PPFD (Tucci et al. 2010).

During the late wet season in our study, the variation in *P*_N_ was similar in both species—the *P*_N_ decreased as PPFD decreased after 13:00. Our results concur with a research on *S. macrophylla* seedlings in northern Brazil, where under well-watered conditions gas exchange decreased as PPFD decreased after 13:00, reaching a minimum at 17:00, whereas *g*_s_ remained low under drought conditions (Cordeiro et al. 2009). Although comparisons of plant physiology between greenhouse and field conditions are limited due to differences in plant age, root depth, and the degree of control of environmental factors (Cordeiro et al. 2009), the similar decreases in *P*_N_ and *g*_s_ measured in the present field study and previous greenhouse studies suggest that stomatal conductance dominates transpiration and strongly affects photosynthesis in both our experimental species.

VPD is an important factor in plant gas exchange, because stomatal opening and closing are strongly dependent on VPD conditions (Cordeiro et al. 2009; Li and Chen 2009; Tucci et al. 2010; Niinemets and Keenan 2014; Chen et al. 2016b). For example, in *Fritillaria cirrhosa*, VPD increased sharply after 10:00, while *g*_s_ dropped rapidly (Li and Chen 2009), suggesting that *g*_s_ in this species is influenced by the environment. A study on *Dialium pachyphyllum* in Cameroon demonstrated similar results in that species (Koch et al. 1994). Here, we found a significant negative relationship between *g*_s_ and VPD_l_ (*P* < 0.01) in *M. azedarach*, which might suggest a mechanism for water loss prevention. We also observed a negative relationship between *g*_s_ and *T*_l_ in *S. macrophylla* during the wet season. Together, these results show that stomata are sensitive to environmental conditions and they close at VPD_l_ above 3 kPa, even in the wet season, in *M. azedarach*. Furthermore, a higher *T*_l_ may be the cause of increased VPD_l_ that is accompanied by lower *g*_s_ and *E* in *S. macrophylla*.

The variation of the leaf area not only reflects the phenology of plantations but also the effects of environmental stress. Thus, there is a strong relationship between LAI and diurnal climate (Hardwick et al. 2015). In *S. macrophylla*, the LAI was reduced only slightly during the dry season, but in *M. azedarach*, more than half of the total leaves fell. Therefore, the different drought tolerance strategies in these species represent the different ways in which they prevent water loss. Arndt et al. (2015) showed that compared to eucalypt savanna, the African mahogany (*Khaya senegalensis*) significantly reduced the total leaf area to moderate water loss in the dry season. Thus, the coordination between plant physiology and leaf area is important for drought adaptation.

Ngugi et al. (2004) reported a non-linear relationship between leaf photosynthesis and *g*_s_ in *Eucalyptus argophloia* and *E. cloeziana*. However, Sun et al. (1995) found a significant linear relationship between leaf photosynthesis and *g*_s_ in drought-resistant *Nothofagus solandri*, and less so in drought-susceptible *N. menziesii*. In our study, *P*_N_ and *g*_s_ in *M. azedarach* exhibited a curvilinear relationship during the wet season, whereas under drought (*g*_s_ < 0.05 mol/m^2^/s), the relationship became linear with a steeper slope than that measured in *S. macrophylla*. This steeper slope demonstrates that *M. azedarach* is more drought-resistant than *S. macrophylla*. Generally, the optimum temperature for photosynthesis ranges from 25 to 30 °C for tropical and subtropical broadleaved trees (Larch 1995). The higher leaf temperatures in *S. macrophylla* compared with those in *M. azedarach* may be the cause of the lower *g*_s_ in warm–wet seasons, leading to lower *P*_N_ (Fig. 5). Additionally, the curvilinear relationship between *C*_i_ and *g*_s_ in *M. azedarach* indicates that *C*_i_ increased with *g*_s_ until a certain CO_2_ concentration was reached, at which point *C*_i_ stabilized whenever *g*_s_ increased. Colom and Vazzana (2003) found similar evidence for water-stressed *Eragrostis curvula*, and studies on *Quercus pubescens* during a dry summer also demonstrated that *C*_*i*_ remains constant due to metabolic limitations or some degree of mesophyll resistance (Haldimann et al. 2008). In *S. macrophylla*, *C*_i_ decreased with increasing *g*_s_ during the dry season, and then increased with *g*_s_ during the wet season, demonstrating the effects of non-stomatal limitations on photosynthesis. Similarly, Koller et al. (2013) found that high *C*_i_ at low *g*_s_ indicates an effect of non-stomatal limitation on photosynthesis in three *Quercus* species. In *M. azedarach*, the negative linear relationship between *g*_s_ and VPD_l_ during the dry season (VPD_l_ > 1.5 kPa) indicates the effects of stomatal limitation on photosynthesis. The same conclusion was made by Zhang et al. (2009) who showed that in dipterocarp species *g*_s_ at a given VPD is significantly reduced during the dry season. Photosynthesis is one of the most temperature-sensitive processes in plants (Yamori et al. 2014). In the wet season, a significant linear relationship between *P*_N_ and *g*_s_ (*P* < 0.01) with different *T*_l_ demonstrated that high temperature in summer months might suppress photosynthesis in *S. macrophylla*. The same conclusion was reached by Slot and Winter (2017), who suggested that the negative effect of rising temperatures on photosynthesis in tropical canopy leaves is almost entirely driven by stomatal processes.

In general, elevated WUE helps plants to adapt to water deficiency in arid regions (Aranda et al. 2007). However, plants with higher WUE demonstrate increased growth during the dry but not the wet season (Picotte et al. 2007). A study conducted in Mediterranean climates (Ogaya and Peñuelas 2003) demonstrated that, compared with mesic species (e.g., *Quercus ilex*), drought-resistant species such as *Phillyrea latifolia* typically experience lower photosynthetic rates and higher transpiration rates, which consequently result in lower WUE. However, during drought conditions, *P*_N_ and WUE in *P. latifolia* can equal or exceed those of *Q. ilex*. During the wet season in the present study, *S. macrophylla* had relatively higher *P*_N_ and WUE values but lower *E* values compared with those in *M. azedarach*. During the dry season, *E* values were very low in both species, but *M. azedarach* had higher WUE than did *S. macrophylla*. This outcome again suggests that *M. azedarach* is a more drought-resistant species when compared to *S. macrophylla*.

Seasonally dry ecosystems such as the Mediterranean or tropical monsoon regions present a challenge for plant growth and survival (Arndt et al. 2015). Noormets et al. (2008) reported that a spring drought might suppress forest canopy development such as leaf sprout and expansion and have long-lasting effects on the forest ecosystem carbon balance. This imbalance remains even when soil water availability improves. In this study, the prolonged drought from October 2009 to April 2010 might have not only decreased leaf photosynthesis but also impaired regional plant productivity. The impact of such stress is represented in the growth performances of *M. azedarach* and *S. macrophylla* (Table 1), where both species showed lower DBH and height compared to those in reported in a previous study in Taiwan (Liao et al. 2011).

## Conclusions

*Melia azedarach* adapted to drought by reducing total leaf area and maintaining higher *P*_N_, *g*_s_, *E*, and WUE compared with those measured in *S. macrophylla* during the dry season. In contrast, *S. macrophylla* had a higher *P*_N_ compared with *M. azedarach* during the wet season and is therefore considered drought-susceptible. In both species, photosynthetic rates during the dry season were affected by multiple, complex environmental factors, but in *S. macrophylla* the temperature had the largest effect on photosynthetic rate during the wet season. However, based on the recovery of *P*_N_ patterns in both species during the morning hours, we propose that both *M. azedarach* and *S. macrophylla* acclimatized to the study site, although they differed in diurnal gas-exchange patterns and drought adaptation strategies. *M. azedarach* has better growth performance under prolonged drought compared with *S. macrophylla*. These findings provide information about photosynthesis under drought conditions that support constructive plantation strategies in tropical areas. More attention should be paid to studying the differences in the photosynthetic responses that are dependent on species and the environment because of the changing climate.
